# Development and validation of the Economic Coercion Scale-20 (ECS-20): A short-form of the ECS-36

**DOI:** 10.1371/journal.pone.0287963

**Published:** 2023-10-19

**Authors:** Stephanie Spaid Miedema, Yuk Fai Cheong, Ruchira Tabassum Naved, Kathryn M. Yount

**Affiliations:** 1 Department of Sociology, Emory University, Atlanta, Georgia, United States of America; 2 Department of Psychology, Emory University, Atlanta, Georgia, United States of America; 3 International Centre for Diarrheal Disease Research (icddr,b), Dhaka, Bangladesh; 4 Hubert Department of Global Health, Rollins School of Public Health, Emory University, Atlanta, Georgia, United States of America; University of Castilla-La Mancha: Universidad de Castilla-La Mancha, SPAIN

## Abstract

The Economic Coercion Scale 36 (ECS-36) is a validated scale measuring women’s exposure to economic coercion for low-income countries. A valid short form is needed to facilitate parsimonious measurement of economic coercion in general surveys or program evaluations. We used data from a probability sample of 930 married women 15–49 years in Matlab, Bangladesh. We selected 21 items from the ECS-36 based on theory, content coverage, and item and dimensional information. We evaluated external validity with measures of non-economic intimate partner violence and depressive symptoms. We tested measurement invariance of the short-form scale across participants and non-participants of microfinance programs. A final, 20-item scale captured husband’s interference with wife’s (1) acquisition of economic resources and (2) use or maintenance of economic resources. IRT results of the ECS-20 demonstrated precision over the higher range of the economic coercion trait. Tests of external validity confirmed expected correlations of the ECS-20 with measures of IPV and depressive symptoms. The ECS-20 was measurement invariant across groups of women who did and did not participate in microfinance programs. The ECS-20, a valid short-form of the ECS-36, is suitable for general surveys and monitoring potential adverse impacts of microfinance programs targeting women.

## Introduction

Worldwide, one in three women report lifetime experience of physical and/or sexual intimate partner violence (IPV) [1], and levels of psychological IPV are similar [2]. These commonly measured forms of IPV, however, may not capture the totality of violence that women experience. Economic coercion against a partner is an understudied form of intimate partner violence against women, particularly in low- and middle-income countries (LMICs) [3–6]. Economic coercion entails efforts to control women’s access to, use of, and maintenance of economic resources [7]. In the United States, the estimated prevalence of economic coercion ranges from more than one third in the general population [8] to more than 90% in shelter populations [9–11]. To date, valid and consistent measurement of economic coercion has been limited, particularly for LMICs [6] despite qualitative evidence that women’s experiences of economic coercion may be common [12, 13]. Valid measures of economic coercion are a particular priority in LMIC settings where women’s economic participation is expanding rapidly.

Bangladesh, a lower middle-income country in South Asia, is a salient site to develop and validate measurement tools to capture the totality of women’s experiences of IPV, including economic coercion. Bangladesh has seen steady economic growth and poverty reduction over the past two decades [14]. The concurrent expansion of export markets and microfinance programs has expanded economic opportunities for women [15]. During 2016–2017, women above age 15 accounted for 30% of the labor force [16]. Yet, growing economic opportunities for women sit in tension with deep-rooted patriarchal family structures and normative expectations of women’s role in the family and community. In Bangladesh, men are expected to serve as head of the household and financial providers, while women provide unpaid domestic and care labor, often under seclusion or with restricted mobility [17]. Gender inequitable attitudes vary geographically but remain common, and men’s perpetration of physical and sexual IPV remains prevalent [18]. In Bangladesh, 73% of ever-married women reported experiencing sexual, physical, economic or emotional violence, or controlling behaviors, by an intimate partner [18]. Among those lifetime experiences, 50% reported ever experiencing physical violence and over one in four (27%) reported experiences of sexual violence [18]. In this context, as women enter or attempt to enter and earn income in the labor market, they may face backlash as they challenge long-standing gender norms [19–21]. For example, a study among female garment workers in Bangladesh found that husbands and other family members sometimes forced women to hand over earned wages, as a way to curtail women’s expanded agency in mobility and access to new resources historically male spaces [22]. In cases where women resisted these economically coercive tactics, they risked facing physical violence [22]. However, in general, research on economic coercion among women in economically transitioning LMICs is limited, in part due to lack of valid measures of economic coercion.

To address this gap in a parent study in Matlab, Bangladesh, entitled *Intimate Partner Coercion and Implications for Women’s Health and Well-being*, the authors here developed a 36-item Economic Coercion Scale (ECS-36), which captures restrictions on women’s access to work, education, and training (14 items) as well as restrictions on women’s use and control over economic resources (22 items) [5]. We developed the ECS-36 using a sequential, mixed-methods design, including formative qualitative research, questionnaire review, cognitive interviewing, a population-based survey, and psychometric validation [5]. The ECS-36 comprehensively measures women’s experiences of two dimensions of economic coercion, namely barriers *to acquire* and *to use or maintain* economic resources. The ECS-36 can be used in studies in which either dimension of economic coercion is a primary outcome. The ECS-36 is especially useful in settings where women’s economic roles have been constrained and are expanding and where participation in microfinance programs is common. However, the use of 36-items for a single scale in household surveys conducted in low-resourced settings may be too long for respondents and survey administrators, particularly in cases where multiple forms of partner violence are among the outcomes assessed. To complement the ECS-36, a short-form version may prove useful in general surveys or impact evaluations that seek to measure multiple primary outcomes. Here, we present findings of a theoretically grounded psychometric analysis to validate a short-form version of the ECS-36.

## Materials and methods

### Sample

The study was conducted in rural Matlab *thana*, Bangladesh, a site 55km southeast of the capital city of Dhaka. The population is majority Muslim and poor, with households typically owning no agricultural land (~50%) and relying on income from remittances (33%), small businesses (24%), and service (21%) [23].

Eligible study participants were married women 15–49 years living with their husbands. The sample was drawn from household censuses of the 142 villages (population 230,000) in the Matlab Health and Demographic Surveillance System (HDSS). A total of 930 of 1015 eligible women (91.6%) were interviewed. The Institutional Review Boards at Emory University (IRB00097428) and International Centre for Diarrheal Diseases Research, Bangladesh (icddr,b) approved the study. The field team followed ethical guidelines for data collection on violence against women, including obtaining informed consent among all participants [24]. Parental or guardian consent was not required for women aged 15–18 in this context. Verbal consent was obtained from all participants prior to initiating the interview. Signatures were required from the interviewer and a witness to confirm the respondent’s verbal consent to participate. Fieldwork occurred from November 2018 to January 2019. Our same project team conducted validation of the original ECS-36 questionnaire and thus no additional permissions were required for modification. Average participant age was 35 years old. On average, participants and their husbands had completed six grades of schooling. Participants were Muslim (89%) and Hindu (11%). A total of 40 economic coercion questions were included in the survey, covering three domains: inhibiting access to work or training; control over money or assets; and economic sabotage. Participates were asked whether each experience had occurred in their lifetime. Participants who answered yes were asked follow-up questions on whether the economically coercive event had occurred in the past 12 months, and if yes, whether it had occurred 1–2 times, 3–5 times, or more than 5 times. Nearly two-thirds of women (62%) experienced any economic coercion in their lifetime, and item-specific lifetime prevalences ranged from 2% (pressured into earing money when she did not want to) to 26% (being told that women should not work outside the home). The ECS-36 was derived through a process of robust psychometric validation. Further details of the sample, survey design, descriptive statistics, and development and validation of the ECS-36 are presented elsewhere [5].

### Item selection

We selected a subset of items from the ECS-36 based on theoretical salience, strength of association with one of the two latent ECS traits, IRT item information (item-level discrimination, difficulties and thresholds, and item characteristic curves), content validity, and maximal coverage of the full range of each latent trait. First, we grouped ECS-36 items according to their estimated difficulty of endorsement along each of the two latent traits, or measured dimensions of economic coercion. Difficulty refers to the level of the latent trait at which the probability of an endorsed response to the item (here, a report for economic coercion = 1) is 0.5. Next, two authors independently selected items from each group based on theoretical salience, content validity, estimated item difficulties, and item precision using item information curves. Item information curves (available upon request) provide information about the precision of a specific item along the underlying latent-trait continuum. Item difficulties were evaluated based on whether the selected items captured variation across the full range of the underlying latent trait. We sought to retain items with higher precision compared to other items with similar estimated difficulties. Items were retained through deliberation and mutual agreement. A final 21 items from the ECS-36 were retained for validation of a short-form scale.

### Analytic strategy

#### Factor analysis

We first conducted exploratory factor analysis (EFA), which is recommended for measures with less prior research, such as the ECS-36 [25]. We tested sequential one-, two-, three-, four-, five and six- factor models on a random one-third split sample (n_1_ = 310), to evaluate model fit across alternative solutions. *A priori* criteria for dropping items included theory, loadings below 0.35, or items with significant cross-loadings greater than or equal to |0.35|. In the best-fitting model, a single item (“has your husband ever made you feel obliged to give him money?”) was dropped due to cross loading. We ran additional sensitivity analysis using a parallel analysis approach to confirm the best-fitting factor structure [26]. We then conducted confirmatory factor analysis (CFA) on a random two-third split sample (n_2_ = 620) to evaluate model fit of the remaining 20-item scale. Use of random split samples enables us to test the factor structure in distinct samples and mitigates the risk that factor structures are due to random chance variation in the data set. We used WLSMV estimation, which is appropriate for categorical data [27], and interpreted factor solutions after GEOMIN (oblique) rotation. Oblique, rather than orthogonal, rotation was used to allow factors to correlate, given expectations based on theory of interdependence between violence-related factors. We used three indices to assess overall fit for the final EFA and CFA models: the comparative fit index (CFI should be ≥ .95), Tucker Lewis Index (TLI should be ≥ .95), and root-mean-square error of approximation (RMSEA should be ≤ 0.06 and 90% CI ≤ 0.06) [28, 29]. All factor analyses accounted for cluster sampling.

#### Item-response-theory (IRT) analysis

We applied IRT methods to assess whether and how observed items, such as responses to questions about economic coercion, were causal expressions of a woman’s location along the continuum of one or more theoretical, unobserved (latent) traits separately [30]. IRT methods are appropriate to reduce a valid long-form scale (such as the ECS-36) to a valid short-form version [31] that captures, as precisely as possible, the desired range of values along each latent-trait continuum being measured.

For this analysis, we compared the fit of a two-parameter versus a one-parameter logistic (2PL vs. 1PL) IRT model. The results of a chi-square test of difference suggested that a two-parameter model with varying slopes had a better fit than a one-parameter model with constant slopes for individual factors (χ^2^(18) = 73.001, p < 0.001). The two-parameter logistic (2PL) IRT model postulates that the probability of endorsing (or responding yes) to an item is a function of the latent trait level of an individual and the difficulty and discrimination of an item [32]. Discrimination refers to an item’s capacity to distinguish respondents at specific levels of the latent trait of economic coercion, with larger values indicating greater discrimination. We used WLSMV theta parameterization (link = probit), which is appropriate for items with binary response options. We first evaluated model fit, using RMSEA, CFI and TLI fit statistics, and IRT-model assumptions of local independence (LI). Assessment of LI evaluates the extent to which the measured latent trait variable is the sole influence on a woman’s response to a given EC item. In other words, for a given woman with a known EC score, her response to one item is independent of her response to any other item. Violating the LI assumption means that model estimates, fit statistics, and derived scores and associated standard errors can be distorted, and thus, differ from the construct being measured [32]. To assess the LI assumption, we examined item residual correlations. Residual correlations greater than |.2| were considered evidence of local dependence [33].

When model assumptions held, we evaluated graphical and numerical item properties. We assessed estimates of the item-level discrimination, difficulties and thresholds, as well as item characteristic curves (ICC). We evaluated item characteristic curves (ICCs) related to the probability of endorsing each response option (e.g., 0 = no EC reported versus 1 = EC reported) for each item as a function of the level of the latent-EC trait. Together, the ICCs allowed us to assess visually the distribution of the location parameters for each item along the latent-trait continuum, and the strength of the relationship between each item and the latent trait (discrimination). In sum, two key IRT assumptions are that, for the individual economic conversion scales, the items measure just one latent trait in common. In addition, conditional upon the latent trait, the responses to the items should be statistically independent.

#### Reliability, external validity and measurement invariance tests

We estimated scale reliability of the two-factor solution using ordinal alpha, ordinal omega and GLB measures, although note that these serve as diagnostic tools as they do not take into account the clustered nature of the data. To determine external validity, based on the final CFA and IRT models, we estimated factor correlations of the ECS-20 with depressive symptoms as measured by a validated 9-item Center for Epidemiologic Studies Depression Scale (CES-D) (34), and a validated unidimensional 19-item measure for physical (6 items), sexual (3 items), and psychological (10 items) IPV, derived from the World Health Organization (WHO) standardized survey questions on IPV in LMICs [34].

Finally, we evaluated the measurement invariance of the two-factor ECS-20 short-form based on women’s participation in microfinance programs. This group variable was chosen, given evidence that participation in microfinance programs may result in economic coercion in the household in LMIC settings [35–37]. Further, evaluation of women’s microfinance participation on economic coercion using the ECS-36 has been published elsewhere [38]. We applied multiple group CFA for binary variables [39]. We estimated configural and scalar models of measurement invariance. Configural models test equivalence of dimensionality, while allowing factor loadings and thresholds to be estimated freely. Scalar models constrain the factor loadings and thresholds to be equal across the two groups. We used χ2, RMSEA, CFI and TLI statistics to evaluate model fit. We evaluated the change in CFI and TLI for nested models. We calculated change in the χ2 and performed a chi-square test of difference to compare configural and scalar models. We used Mplus 8.0 [39] for all statistical analyses.

## Results

### Factor analysis

Table 1 presents model estimates and fit statistics for the final EFA (n_1_ = 310) and CFA (n_2_ = 620) of the short-form ECS. A final, best-fitting two-factor model was selected using EFA, based on a priori criteria including fit statistics, item loadings and cross-loadings, and theory and contextual salience of items. Results of additional sensitivity analysis using parallel analysis supported the two-factor solution. In EFA, a single item (“made you feel obliged to give him money”) was dropped due to cross loading. Estimated factor loadings were generally high in the final 20-item EFA and CFA models. EFA factor loadings ranged from 0.350–0.990 (Factor 1) and from 0.514–0.941 (Factor 2). CFA factor loadings ranged from 0.492–0.918 (Factor 1) and from 0.611–0.892 (Factor 2). Model fit statistics were within the recommended ranges. Factor correlations were 0.258 in the final EFA and 0.499 in the final CFA.

**Table 1:** Model estimates and fit statistics from exploratory factor analysis (EFA) (n_1_ = 310) and confirmatory factor analysis (CFA) (N_2_ = 620), using geomin rotation for items in the Economic Coercion Scale (ECS) short form, married women 16–49 years living with their husbands in Matlab, Bangladesh, 2018–2019.

		EFA		CFA	
Item # (item # from ECS-36)^§^	Item Label	Factor 1	Factor 2	Factor 1	Factor 2
**1 (1)**	disallowed you to go to your work, school or training, or do any home-based income earning activity	0.990*		0.918*	
**2 (2)**	told you that you could work outside the home only if you kept up with the housework	0.732*		0.612*	
**3 (3)**	told you that you could earn income only if you worked from home	0.736*		0.717*	
**4 (4)**	been wary that you might meet other men when you leave the house for work, school or training	0.846*		0.722*	
**5 (7)**	threatened to hurt you or your children, or threatened to throw you out or abandon you if you worked	0.810*		0.647*	
**6 (9)**	told you that women shouldn’t work outside the home?	0.650*		0.68*	
**7 (12)**	refused to allow you to continue any education or training after marriage	0.946*		0.748*	
**8 (31)**	forbidden you from becoming a microcredit member, group savings member, or opening a bank account	0.350*		0.492*	
**9 (32)**	demanded that you quit your job, schooling or training	0.882*		0.916*	
**10 (14)**	hidden money so that you could not find it		0.941*		0.859*
**11 (17)**	made you fear the consequences if you asked him for money		0.803*		0.892*
**12 (19)**	made you ask him for money for special purchases, such as cosmetics, sari/dresses or special food for guests		0.787*		0.783*
**13 (20)**	refused to give you money to buy food, clothes or other necessities, even when he had the money		0.791*		0.845*
**14 (21)**	blown through/spoiled money despite household needs		0.931*		0.833*
**15 (23)**	decided how you should spend money rather than letting you spent it how you saw fit		0.565*		0.681*
**16 (25)**	made important financial decisions without talking with you about them first		0.514*		0.735*
**17 (27)**	taken your money from you without your permission or knowledge		0.793*		0.738*
**18 (30)**	beaten you up if you challenged his financial decisions		0.725*		0.74*
**19 (39)**	pawned or sold your own or your shared belongings or property without your knowledge or consent		0.562*		0.611*
**20 (40)**	not given you money so that you had to take out loans to cover household expenses		0.733*		0.734*
**F1-F2 Correlation**		0.258*		0.499*	
**Chi-square (df)**		177.7549(154)		278.945* (169)	
**CFI**		0.975		0.947	
**TLI**		0.969		0.941	
**RMSEA**		0.024		0.032	

RMSEA = root mean square error of approximation; CFI = comparative fit index; TLI = Tucker-Lewis index

*Significant at the 5% level

^§^Original ECS-36 initial item pool included more than 36 items.

### IRT analysis

We evaluated the residual correlations to assess local independence within the two parameter multi-dimensional correlated trait IRT model. We identified 27 or 14.21% of the 190 possible item pairs (20*(20–1)/2) as possible violations of local independence. Among the 27 item pairs, six had residual correlations greater than |.20|. The pairs included nine items, including item 3 (“told you that you could earn income only if you worked from home”), item 7 (“refused to allow you to continue any education or training after marriage”), item 10 (“hidden money so that you could not find it”), item 11 (“made you fear the consequences if you asked him for money”), item 15 (“decided how you should spend money rather than letting you spend it how you saw fit”), item 18 (“beaten you up if you challenged his financial decisions”), item 8 (“forbidden you from becoming a microcredit member, group savings member, or opening a bank account”), item 19 (“pawned or sold your own or your shared belongings or property without your knowledge or consent”) and item 20 (“not given you money so that you had to take out loans to cover household expenses”). Among these common items, the item-level distribution ranged from 2.69% (item 19 “pawned or sold your own or your shared belongings or property without your knowledge or consent”) to 12.59% (item 11 “made you fear the consequences if you asked him for money”). However, as items were considered theoretically salient, they were retained.

Table 2 provides item-level discrimination and difficulty estimates. For Factor 1 (barriers to access of economic resources), item-level discrimination (*a*_*1*_) from high to low ranged from 2.317 (“disallowed you to go to your work, school or training, or do any home-based income earning activity”) to 0.565 (“forbidden you from becoming a microcredit member, group savings member, or opening a bank account”). Item-level endorsement (*c*) ranged from a high of -0.886 (“told you that women shouldn’t work outside the home”) to a low of -2.588 (“threatened to hurt you or your children, or threatened to throw you out or abandon you if you worked”). For Factor 2 (control over use of economic resources), item-level discrimination (*a*_*2*_) ranged from 1.978 to 0.771. Endorsement estimates ranged from -1.196 (“decided how you should spend money rather than letting you spent it how you saw fit”) to -2.697 (“blown through/spoiled money despite household needs”). Item characteristic curves are available upon request.

**Table 2:** Item-level estimates (slopes, a_1_ and a_2_, and thresholds, c) for correlated-trait IRT model, married women 16–49 years living with their husbands, Matlab, Bangladesh, 2018–2019 (n_2_ = 620).

Item # (item # from ECS-36)	Item Label	a_1_	s.e.	a_2_	s.e.	c	s.e.
**1 (1)**	disallowed you to go to your work, school or training, or do any home-based income earning activity	2.317	0.524	0	-----	-2.366	0.437
**2 (2)**	told you that you could work outside the home only if you kept up with the housework	0.774	0.129	0	-----	-1.968	0.146
**3 (3)**	told you that you could earn income only if you worked from home	1.028	0.136	0	-----	-1.852	0.174
**4 (4)**	been wary that you might meet other men when you leave the house for work, school or training	1.043	0.161	0	-----	-1.933	0.197
**5 (7)**	threatened to hurt you or your children, or threatened to throw you out or abandon you if you worked	0.849	0.216	0	-----	-2.588	0.251
**6 (9)**	told you that women shouldn’t work outside the home?	0.929	0.103	0	-----	-0.886	0.12
**7 (12)**	refused to allow you to continue any education or training after marriage	1.127	0.137	0	-----	-2.389	0.16
**8 (31)**	forbidden you from becoming a microcredit member, group savings member, or opening a bank account	0.565	0.138	0	-----	-1.758	0.127
**9 (32)**	demanded that you quit your job, schooling or training	2.277	0.45	0	-----	-3.254	0.482
**10 (14)**	hidden money so that you could not find it	0	-----	1.682	0.228	-2.177	0.197
**11 (17)**	made you fear the consequences if you asked him for money	0	-----	1.978	0.37	-2.44	0.384
**12 (19)**	made you ask him for money for special purchases, such as cosmetics, sari/dresses or special food for guests	0	-----	1.257	0.14	-1.904	0.182
**13 (20)**	refused to give you money to buy food, clothes or other necessities, even when he had the money	0	-----	1.581	0.308	-2.432	0.33
**14 (21)**	blown through/spoiled money despite household needs	0	-----	1.507	0.229	-2.697	0.286
**15 (23)**	decided how you should spend money rather than letting you spent it how you saw fit	0	-----	0.93	0.116	-1.196	0.124
**16 (25)**	made important financial decisions without talking with you about them first	0	-----	1.082	0.178	-1.63	0.193
**17 (27)**	taken your money from you without your permission or knowledge	0	-----	1.094	0.214	-2.616	0.254
**18 (30)**	beaten you up if you challenged his financial decisions	0	-----	1.1	0.175	-2.398	0.238
**19 (39)**	pawned or sold your own or your shared belongings or property without your knowledge or consent	0	-----	0.771	0.19	-2.424	0.23
**20 (40)**	not given you money so that you had to take out loans to cover household expenses	0	-----	1.08	0.2	-2.419	0.272

### External validity and measurement invariance tests

Reliability tests demonstrated strong reliability of the short-form scale (ordinal alpha = 0.88; ordinal omega = 0.88; GLB greatest lower bound = 0.94). Tests of external validity confirmed expected correlations of the ECS-20 with measures of IPV and depressive symptoms (Table 3). ECS-20 Factor 1 was moderately correlated with a unidimensional measure of psychological, physical and sexual IPV (r = 0.359, SE = 0.032) and weakly correlated with a 9-item modified CES-D scale measuring depressive symptoms in the past two weeks (r = 0.237, SE = 0.055). ECS-20 Factor 2 was highly correlated with measures of IPV (r = 0.732, SE = 0.018) and depressive symptoms (r = 0.503, SE = 0.045). Correlations were in the expected directions for all factors.

**Table 3:** Correlations between two factor economic coercion scale- short form, unidimensional measure of intimate partner violence and 9-item center for epidemiologic depression scale.

	Economic Coercion SF Factor 1	Economic Coercion SF Factor 2	Unidimensional IPV	9-item CES-D
**Economic Coercion SF Factor 1**	-			
**Economic Coercion SF Factor 2**	0.464 (0.038)	-		
**Unidimensional IPV**	0.359 (0.032)	0.732 (0.018)	-	
**9-item CES-D**	0.237 (0.055)	0.503 (0.045)	0.346 (0.027)	-
χ^2^ (df) = 1569.620* (1076); RMSEA = 0.022; CFI = 0.956; TLI = 0.954				

RMSEA = root mean square error of approximation; CFI = comparative fit index; TLI = Tucker-Lewis index

Tests for measurement invariance demonstrated scalar invariance of the ECS-20 between women who did and did not participate in microfinance programs (Table 4). The configural (RMSEA = 0.028, CFI = 0.962, TLI = 0.957) and scalar (RMSEA = 0.027, CFI = 0.963, TLI = 0.960) models showed good fit. We found minimal difference between fit statistics between models (ΔRMSEA = -0.001, ΔCFI = 0.001, ΔTLI = 0.003). We used the difftest command in Mplus, which is appropriate for weighted least squares means and variance adjusted estimates to test differences in model fit [39]. The chi-square based on the Mplus diff test feature showed no significant difference between models (Δχ2 = 20.080, *p* = 0.2167), thereby indicating configural and scalar measurement invariance of the ECS-20 between the two groups. Fit statistics for each of the two subsamples are available upon request.

**Table 4:** Results of invariance test for the two-factor model of the 20-item Economic Coercion Scale (ECS) short form.

Factor Model	WLSMV-χ2	*df*	RMSEA	ΔRMSEA	CFI	ΔCFI	Contrast	Δχ2	*df*
A. Configural measurement invariance	464.930 *	338	0.028		0.962				
B. Scalar measurement invariance	476.041*	354	0.027	0.001	0.963	-0.001	B vs. A	20.080	16

RMSEA = root mean square error of approximation; CFI = comparative fit index; Δχ2 = chi-square based on difftest

## Discussion

Microfinance programs designed to empower women economically may inadvertently increase women’s risk of economic coercion, and other forms of IPV, although the evidence is mixed [35, 40–42]. The lack of validated scales to measure economic coercion has hampered the ability to monitor and to evaluate the impact of women’s greater economic opportunities on women’s risk of economic coercion. To address this gap in the literature, we have validated the ECS-20, a short-form of the ECS-36 [5], using a theoretically grounded, psychometric analytic approach.

As with the ECS-36, the ECS-20 captures two dimensions of economic coercion: (1) husband’s interference with wife’s acquisition of economic resources, such as training or paid work and (2) husband’s interference with wife’s use or maintenance of economic resources. The factor structure of the ECS-20 also parallels that of the ECS-36. IRT results similarly demonstrated precision over the higher range of the economic coercion trait for each dimension. As with the ECS-36 [43], tests of external validity confirmed expected positive correlations of the ECS-20 with measures of IPV, consistent with the idea that economic coercion is a distinct, yet correlated form of IPV. To test further external validity of the ECS-20, we evaluated correlations with depressive symptoms and found those to be in the expected direction. Tests for measurement invariance demonstrated scalar invariance between women who did and did not participate in microfinance programs. Scalar invariance indicates strong factorial invariance. In other words, the factor structure, loadings and thresholds of the ECS-20 are equal between both groups of women. These results suggest that the ECS-20 can be used in the same manner (i.e., without modification) among all women–irrespective of their participation in microfinance programs.

### Research implications

Measurement of economic coercion is in its “infancy” worldwide, and particularly in LMICs [6]. The ECS-20 provides a short form of the ECS-36 to measure women’s experiences of economic coercion in LMICs. The ECS-20 will be useful for impact assessments of microfinance programs when multiple primary outcomes are being assessed. For example, qualitative research conducted under the parent study shows how inequitable gendered structures inhibit women’s agentic decision-making and control over microfinance loans in this context [37]. The ECS-20 could be a useful tool to assess the ways in which microfinance programs may shift patterns of economic coercion among women in rural Bangladesh. We also encourage use of the ECS-36 for comprehensive measurement of economic coercion, and the ECS-20 when survey constraints do not allow full measurement. We also acknowledge the practical limitations of retaining all 20 items in some multipurpose general surveys, like the Demographic and Health Surveys (DHS), suggesting that a third, ‘screener’ ECS scale would be a useful complement in the family of ECS scales. The bi-dimensionality of the ECS-36 and the ECS-20 enables researchers to select scale dimensions that are relevant to the local context [5]. For example, in settings where women’s access to work, training and education is widespread and normative, researchers may choose to omit Factor 1 (barriers to access economic opportunities) and prioritize Factor 2 (control over economic resources). Finally, the ECS-20, or selected subscales, may be a useful option in the family of economic coercion scales for monitoring sustainable development goal 5.2 (SDG5.2), to eliminate all forms of violence against women.

### Limitations

This study faced some limitations. While the ECS-36 and ECS-20 are intended for wider use across LMIC contexts, both scales were developed and validated in rural Bangladesh. Replication of the parent study in urban areas and other LMICs is needed to validate both scales more widely. Further validation among women in LMICs from diverse religious, economic and social backgrounds will help to confirm the applicability of the ECS-20 in widely diverse populations. Second, while the theoretically informed empirical process of generating the ECS-20 sought to minimize reductions in prevalence from dropping EC items, the ECS-36 captures more comprehensively the prevalence of economic coercion. In the same sample of women in this study, the lifetime prevalence of women’s experiences of economic coercion was estimated to be 62.26% using the ECS-36 [5] and was 54.19% using the ECS-20. As a result, we recommend use of the ECS-36 when the intent is to capture fully the prevalence of economic coercion and use of the ECS-20 when the intent is to assess impact of microfinance interventions on multiple dimensions of women’s lives and well-being with more parsimonious scales. Further, an even shorter version of the ECS-36 may be useful to screen for economic coercion in clinical settings or in multipurpose national surveys, like the DHS.

## Conclusions

In conclusion, the ECS-20 is a valid short-form of the ECS-36 and is suitable to assess potential adverse impacts of microfinance programs that target women. Standard application of the ECS-20 will allow comparative impact evaluation of different microfinance interventions to identify highly effective intervention approaches. Overall, use of the family of ECS scales will enable us to have a more encompassing understanding of women’s experiences of IPV.

## Supporting information

S1 Dataset (DTA)

S1 File Inclusivity in global research. (DOCX)
